# A Novel Molecular Signature of Cancer-Associated Fibroblasts Predicts Prognosis and Immunotherapy Response in Pancreatic Cancer

**DOI:** 10.3390/ijms24010156

**Published:** 2022-12-21

**Authors:** Weiyu Ge, Ming Yue, Yanling Wang, Yongchao Wang, Shengbai Xue, Daiyuan Shentu, Tiebo Mao, Xiaofei Zhang, Haiyan Xu, Shumin Li, Jingyu Ma, Liwei Wang, Jiujie Cui

**Affiliations:** State Key Laboratory of Oncogenes and Related Genes, Shanghai Cancer Institute, Department of Oncology, Renji Hospital, School of Medicine, Shanghai Jiao Tong University, Shanghai 200127, China

**Keywords:** cancer-associated fibroblasts, molecular signature, tumor immune microenvironment, therapeutic sensitivity, pancreatic cancer

## Abstract

Cancer-associated fibroblasts (CAFs), a prominent population of stromal cells, play a crucial role in tumor progression, prognosis, and treatment response. However, the relationship among CAF-based molecular signatures, clinical outcomes, and tumor microenvironment infiltration remains largely elusive in pancreatic cancer (PC). Here, we collected multicenter PC data and performed integrated analysis to investigate the role of CAF-related genes (CRGs) in PC. Firstly, we demonstrated that α-SMA^+^ CAFs were the most prominent stromal components and correlated with the poor survival rates of PC patients in our tissue microarrays. Then, we discriminated two diverse molecular subtypes (CAF clusters A and B) and revealed the significant differences in the tumor immune microenvironment (TME), four reported CAF subpopulations, clinical characteristics, and prognosis in PC samples. Furthermore, we analyzed their association with the immunotherapy response of PC patients. Lastly, a CRG score was constructed to predict prognosis, immunotherapy responses, and chemosensitivity in pancreatic cancer patients. In summary, these findings provide insights into further research targeting CAFs and their TME, and they pave a new road for the prognosis evaluation and individualized treatment of PC patients.

## 1. Introduction

The poor prognosis of pancreatic cancer (PC) urges us to more deeply understand its potential molecular mechanism and seek better therapies. Cancer-associated fibroblasts (CAFs), a significant fraction of the pancreatic cancer stroma, contribute to a dense stromal accumulation in PC [1,2]. Previous studies have demonstrated that CAFs can facilitate the malignant phenotypes of tumors, particularly tumorigenesis and invasion, inflammation, and extracellular matrix (ECM) remodeling [3,4]. As the leading participant of the desmoplastic stroma in PC, CAFs play a crucial part in diverse clinical responses, drug tolerance, and the tumor immunosuppressive environment by producing ECM proteins and cytokines and interacting with cancer cells [5,6,7,8]. The intratumoral heterogeneity of CAFs in the stroma of PC has been extensively studied. Four major distinct subpopulations of CAFs have been demonstrated in PC: (1) myCAF, the myofibroblastic subset (myCAF) characterized by smooth muscle actin expression, high transforming growth factor (TGF) signaling, and ECM components [1,9]; (2) iCAF, the inflammatory subset characterized by high expressions of inflammatory mediators [10]; (3) apcCAF, the antigen-presenting subset characterized by the expression of CD74 and MHC class II [11]; and (4) meCAF, the highly activated metabolic subset characterized by high expression of PLA2G2A and CRABP2 [12].

Some CAF markers have been studied separately in the past few years, such as α-smooth muscle actin (α-SMA), fibroblast activation protein, CD29, fibroblast-specific protein 1, platelet-derived growth factor receptor B, and podoplanin [13]. For instance, tumors accumulated with the α-SMA^+^ fibroblasts have worse prognoses and higher invasiveness, and they can affect therapeutic reactions [13,14]. Fibroblast activation protein-positive CAFs can lead to immunosuppression and resistance to immunotherapy [15]. However, whether the CAF-mediated tumor microenvironment (TME) is associated with tumor characteristics and the underlying molecular mechanism remains unclear [16,17]. In addition, the guiding significance of current pathological and molecular classification for PC treatment is limited [18]. Although existing strategies targeting the stroma have suppressed tumor growth and enhanced treatment responses in the mouse model, clinical trials have not yet produced promising results [6,19]. Some of these strategies even lead to tumor recurrence and metastasis [20], which suggests that accurate identification of the CAF molecular subtypes in PC is necessary to apply stromal-targeting therapies efficiently.

This study determined that PC patients with accumulated α-SMA^+^ CAFs had a poor prognosis regarding tissue microarrays, and that myCAF, apcCAF, and meCAF subsets were highly enriched in PC. Then, we thoroughly estimated the expression profiles of CAF-related genes (CRGs) and their influences on prognosis, clinical features, and immune cell infiltration in PC patients. Furthermore, we constructed a CRG score to predict PC patients’ prognoses, clinical outcomes, immunotherapy responses, and chemosensitivity. Our findings could deepen our understanding of CRGs and smooth the way for prognosis evaluation and personalized therapy strategies in PC patients.

## 2. Results

### 2.1. α-SMA^+^ CAFs Accumulate in PC Tissues with Worse Prognoses

**Figure 1:**
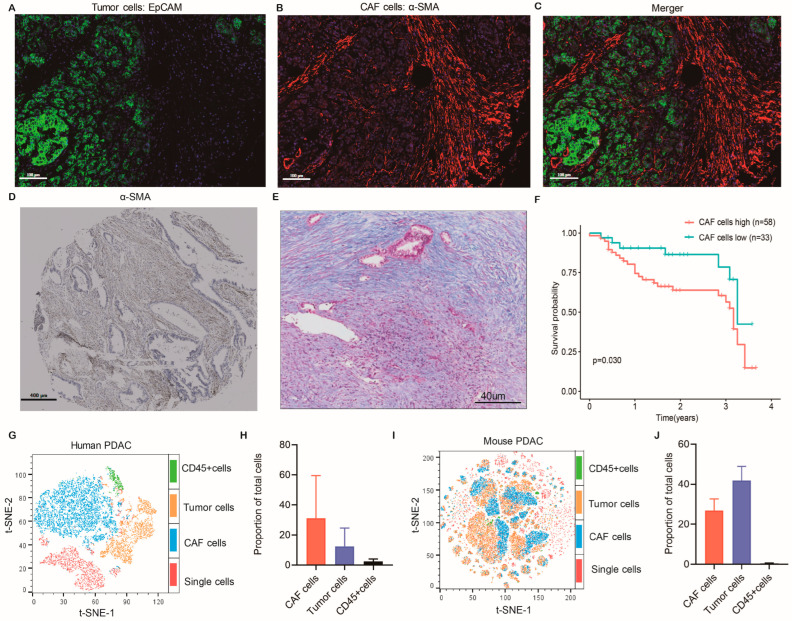
Pancreatic cancer (PC) tissues accumulating α-smooth muscle actin (α-SMA)-positive cancer-associated fibroblasts (CAFs) show a poor prognosis. (**A**–**C**) Detection of α-SMA and EpCAM using double immunofluorescence to differentiate CAFs and epithelial cell populations in human PC tissue microarrays. (**D**) Representative images of α-SMA immunohistochemistry staining in human PC tissue microarray. (**E**) Representative Masson staining in human PC tissues. Blue color: stroma. (**F**) Human PC tissues were classified into α-SMA-high or α-SMA-low CAF groups on the basis of α-SMA immunohistochemistry score, followed by examining patients’ overall survival using Kaplan–Meier survival analysis by log-rank test. (**G**,**H**) The t-SNE plot of CAFs (CD45^−^EpCAM^−^CD29^+^), tumor cells (CD45^−^CD29^−^EpCAM^+^), and tissue leukocytes (CD29^−^EpCAM^−^CD45^+^) were measured by flow cytometry in human PC tissues. (**I**,**J**) The t-SNE plot of CAFs (CD45^−^EpCAM^−^PDPN^+^), tumor cells (CD45^−^PDPN^−^EPCAM^+^), and tissue leukocytes (PDPN^−^EpCAM^−^CD45^+^) were measured by flow cytometry in mouse-derived allograft tissues.

**Figure 2:**
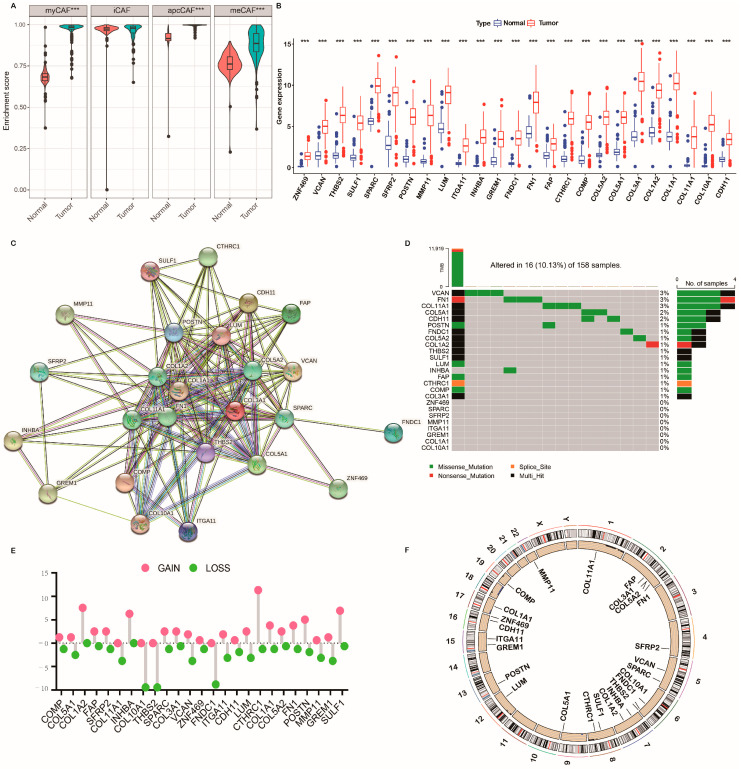
Genetic and transcriptional alterations of CAF-related genes (CRGs) in PC. (**A**) The enrichment scores of four CAF subsets between normal and PC tissues. (**B**) Expression distributions of 25 CRGs between normal and PC tissues from GTEx and TCGA cohorts. (**C**) The protein–protein interaction network acquired from the STRING database among the CRGs. (**D**) Mutation frequencies of CRGs in 158 PC patients from TCGA cohort. (**E**) Frequencies of copy number variation (CNV) gain, loss, and non-CNV among CRGs, pink and green represent gain and loss of CNV, respectively. (**F**) Locations of CNV alterations in CRGs on 23 chromosomes. *** *p* < 0.001 and not significant (*p* > 0.05) according to repeated-measures Wilcoxon test.

Previous studies have shown that α-SMA is a marker of activated CAFs and an efficacy evaluation indicator of targeted CAF therapy [21,22]. The Kaplan–Meier curves showed worse overall survival (OS) in patients with high α-SMA^+^ CAFs accumulated in PC (Figure 1F). Immunofluorescence staining, immunohistochemistry, and Masson staining were used to further confirm the CAF population in PC tissue microarrays (Figure 1A–E). The results explain that α-SMA^+^ CAFs, as a prominent desmoplastic stroma, were remarkably enriched in PC tissues. To define the substantial proportion of CAFs in human PC tissues and mouse-derived allografts, we excluded hematopoietic and epithelial cells using flow cytometry analysis with CD45 and EpCAM markers. We also identified CAFs using human fibroblast markers (integrin b1/CD29) and mouse fibroblast marker (PDPN). The fresh human samples included PC patients at the time of surgery before any treatment. Figure 1G–J illustrate that the CAFs accounted for about 30% of all cellular populations in human and mouse tumor tissues. We further assessed the enrichment scores of four reported CAF subtypes in pancreatic tumors and normal pancreas samples from TCGA-PAAD and GTEx cohorts, and we found that myCAF, apcCAF, and meCAF were abundant in the tumor samples (Figure 2A). These results suggest that CAFs are essential components of TME in PC, which may modulate tumorigenesis and progression.

### 2.2. Genetic Mutation Landscape of CRGs in PC

We first determined the expression levels of the 25 CRGs in tumor specimens and normal specimens, and we observed that almost all CRGs were abundant in the tumor specimens (Figure 2B). To reveal the interaction of CRGs, we performed a PPI analysis. Figure 2C displayed that COL1A1, COL11A1, COL3A1, COL5A2, COL1A2, FN1, FAP, CDH1, POSTN, COMP, COL5A1, COL10A1, and THBS2 were hub genes. Furthermore, we identified the total frequency of somatic mutations and copy number variations (CNVs) of the 25 CRGs in PC. As depicted in Figure 2D, 16 of 158 (10.13%) PC samples emerged with genetic mutations. Figure 2D also indicated that, among the 25 CRGs, VCAN, FN1, and COL11A1 were the genes with the highest mutation rate, followed by COL5A1 and CDH1. In addition, we demonstrated evident CNV alterations of the 25 CRGs (Figure 2E). We also analyzed the CNV alteration location of the 25 CRGs on chromosomes using the “circlize” R package (Figure 2F). We concluded that CNV might act in regulating the expression of 25 CRGs. These findings reveal significant differences in the genomic background and expression levels of the 25 CRGs between PC and normal samples, implying the latent roles of the 25 CRGs in PC tumor progression.

### 2.3. Identification of CAF Subtypes and Characteristics of the TME in PC

**Figure 3:**
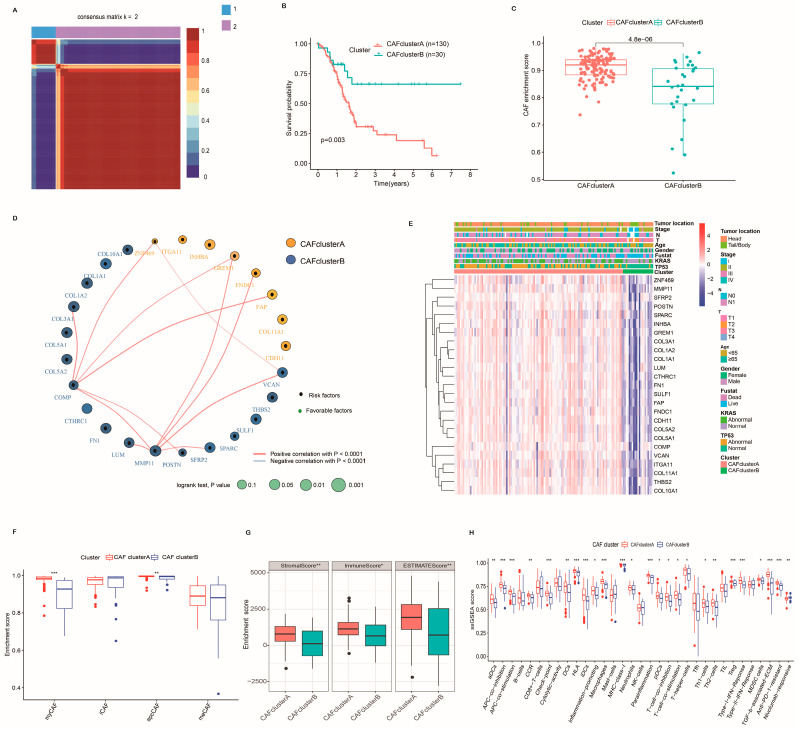
Identification of CAF subtypes and characteristics of the TME in PC. (**A**) Consensus matrix heatmap defining two clusters (k = 2) and their correlation area. (**B**) Kaplan–Meier plot of overall survival (OS) by CAF clusters for PC patients in TCGA cohort (*p* = 0.003, log-rank test). (**C**) Box plots showing CAF enrichment score between CAF cluster A and CAF cluster B. (**D**) A network of correlations including CRGs in TCGA cohort. (**E**) Differences in clinical features and expression levels of CRGs between the two distinct subtypes. Stage, gender, age, survival status, and cluster were used as patient annotations. (**F**) The enrichment score of four CAF subsets between CAF cluster A and CAF cluster B. (**G**) Correlations between the two CAF clusters and TME score. (**H**) The infiltration abundance of 33 TME cells of two CAF subtypes in PC. The Wilcoxon test analyzed the statistical differences between the two clusters (*** *p* < 0.001, ** *p* < 0.01, * *p* < 0.05, and not significant (*p* > 0.05)).

To better understand the expression pattern of CRGs in tumorigenesis, we performed a subsequent analysis of 160 PC patients from TCGA-PAAD. Table S2 lists detailed information about these patients. We further performed a consensus clustering analysis to investigate the relationships between the expression pattern of CRGs and PC subtypes, and we classified PC patients according to the expression levels of these CRGs. Our findings indicate that k = 2 is an optimal choice to divide the entire cohort into CAF cluster A (*n* = 130) and CAF cluster B (*n* = 30) (Figure 3A and Figure S1). Moreover, we used the ICGC cohort to verify the repeatability of the clustering. We also conducted a consensus clustering analysis on this cohort and classified the cohort into two distinct subtypes (Figure S2A,B). Patients with CAF cluster A had worse OS than patients with CAF cluster B in both TCGA and ICGC cohorts (Figure 3B and Figure S2C). We further dissected the CAF signature of the patients in two CAF subtypes. The expression of the CAF signature in CAF cluster A was substantially higher than in cluster B (Figure 3C). Figure 3D presents the relevant networks of CRG interactions and regulator connections. It also illustrates the prognostic value of CRGs (Table S3) and the enrichment of the CRG-related KEGG pathways (Table S4) in PC patients. Additionally, significant differences in the genomic expression of CRGs and clinical variables were observed between the two CAF clusters (Figure 3E).

Apart from the differences in prognosis and genome between CAF cluster A and CAF cluster B, there were also distinct discrepancies in immune cell infiltration and TME score between them. Firstly, we observed higher enrichment scores of myCAF and apcCAF in the CAF cluster A group (Figure 3F). To investigate the roles of CRGs in the TME of PC, we then evaluated the association among the two CAF clusters, 33 immune cell subtypes, and the TME score (Table S5). Compared with CAF cluster B, CAF cluster A had higher immune and stromal scores (Figure 3G) and higher infiltration levels of immunosuppressive cells, such as regulatory T cells (Tregs), MDSC cells, and DC cells, and other immunosuppressive factors, such as TGF-β-associated ECM (Figure 3H). More importantly, we detected a higher enrichment score of anti-PD-1-resistant signatures and a lower enrichment score of nivolumab-responsive signatures in CAF cluster A (Figure 3H), indicating that patients in the CAF cluster A group may be less sensitive to immunotherapy. These results imply that the CAF cluster A group may be closely associated with stromal activation and immunosuppression features.

### 2.4. Establishment and Verification of the Prognostic CRG Score

The CRG score was created according to the LASSO and multivariate Cox (multiCox) analysis for 25 CRGs. Eventually, we obtained five hub genes (*VCAN*, *COL1A2*, *ZNF469*, *SPARC*, and *FNDC1*). The CRG score was calculated as follows:

$$\begin{array}{l} \mathrm{CRG}\;\mathrm{score}=\left(0.437\times \mathrm{expression}\;\mathrm{of}\;VCAN\right)+\left(1.33\times \mathrm{expression}\;\mathrm{of}\;COL1A2\right)\\ \;\;\;\;\;\;+\left(-0.807\times \mathrm{expression}\;\mathrm{of}\;ZNF469\right)+\left(-1.282\times \mathrm{expression}\;\mathrm{of}\;SPARC\right)\\ \;\;\;\;\;\;+\left(-0.262\times \mathrm{expression}\;\mathrm{of}\;FNDC1\right). \end{array}$$

**Figure 4:**
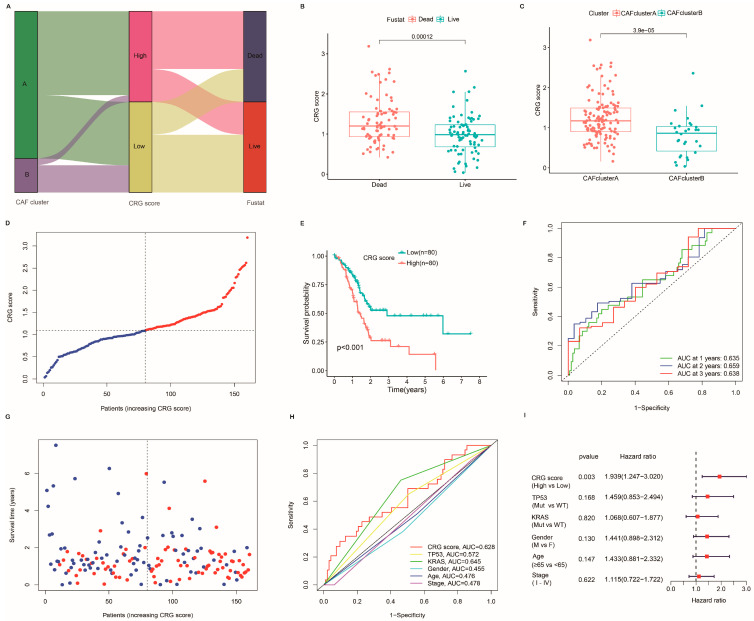
Establishment of the CRG score in TCGA cohort. (**A**) Alluvial diagram of subtype distributions in groups with distinct CRG score and survival status. (**B**) CRG score was significantly elevated in patients who died during follow-up. (**C**) Box plots displaying discrepancies in CRG scores between the two CAF subtypes. (**D**,**G**) Ranked dot and scatter plots showing the CRG score distribution and survival outcomes. Red and blue represent dead and alive of PC patients, respectively. (**E**) Kaplan–Meier plot of overall survival of patients with high and low CRG scores (*p* < 0.001, log-rank test). (**F**) Receiver operating characteristic curves to predict the sensitivity and specificity of 1-, 2-, and 3-year survival according to the CRG score. (**H**) Time-dependent receiver operating characteristic curves of the nomograms compared for 1-, 2-, and 3-year OS in PC, respectively. (**I**) Multivariate Cox regression analysis demonstrated that CRG score was the most critical risk factor for OS in PC among clinical factors.

Figure 4A displays the distribution of patients in the two CAF clusters and two CRG-score groups. Compared with alive patients, the CRG score was significantly elevated in patients who died during follow-up (Figure 4B), and CAF subtype A had higher CRG scores (Figure 4C). The risk plot of the CRG score indicated that, with an increasing CRG score, OS time decreased while mortality rose (Figure 4D,G). Patients with higher CRG scores in both categories were associated with worse survival rates (Figure 4E). Additionally, the AUC values of 1-, 2-, and 3-year OS were 0.63, 0.659, and 0.638, respectively (Figure 4F). Moreover, the CRG score retained excellent predictability in assessing the prognosis of PC patients (Figure 4H). Among multiple clinical features, multivariate Cox regression modeling proved that the CRG score was the only independent risk factor for the OS of PC patients in the TCGA cohort (Figure 4I).

### 2.5. Characteristics of the TME and Function Enrichment in Distinct Subgroups

**Figure 5:**
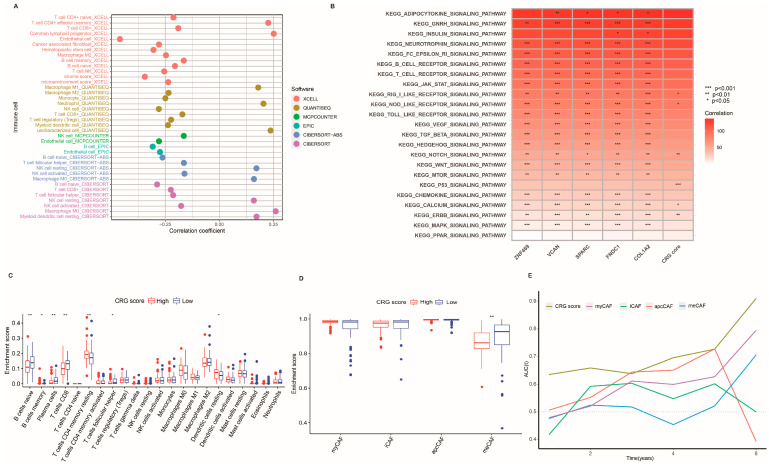
Association of CRG scores with the immune microenvironment. (**A**) Correlations between the CRG score and immune infiltration. (**B**) GSVA performed in CRG score signature based on TCGA. (**C**) The infiltration abundance of 22 TME cells of two CRG-score groups in PC. (**D**) The enrichment score of four CAF subsets between the low-CRG-score group and the high-CRG-score group. (**E**) tROC analysis showed that the GRC score was an accurate variable for survival prediction. The Wilcoxon test analyzed the statistical differences between the two clusters (** *p* < 0.01 and not significant (* *p* > 0.05)).

To examine the association between CRG score and the TME of PC, we analyzed their immune microenvironment in detail. As confirmed by different methods, the CRG score was positively associated with M1 macrophages and neutrophils, whereas it was negatively related to B cells, NK cells, CD8 T cells, and CD4 T cells (Figure 5A). Moreover, we sought to explore the potential pathways related to the CRG score using GSVA. Several cancer-associated pathways (P53, Notch, and ERBB pathways) were most closely correlated with the CRG score (Figure 5B). Consistently, we found that the enrichment levels of B cells, plasma cells, CD8 T cells, and CD4 T cells were markedly higher in the low-CRG-score group than in the high-CRG-score group (Figure 5C). Figure 5D reveals a higher enrichment score of meCAF in the low-CRG-score group. Furthermore, time-dependent receiver operating characteristic (tROC) analysis showed that the CRG score was the most accurate predictor for overall survival compared with other single-CAF subsets in PC (Figure 5E). These findings indicate that patients with lower CRG scores had higher meCAF accumulation and more immune cell infiltration.

### 2.6. Association of the CRG Score with Tumor Mutation Burden (TMB) and Mutation

**Figure 6:**
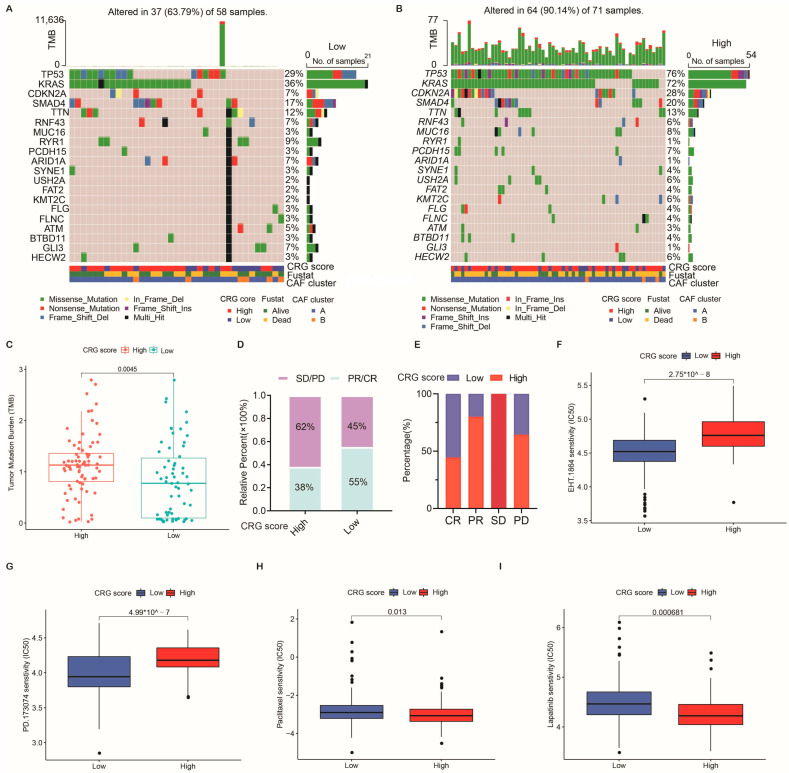
Relationships among the CRG score, tumor mutation, and therapeutic susceptibility in PC. (**A**,**B**) Waterfall plot of somatic mutation properties according to low and high CRG scores. (**C**) TMB in diverse CRG-score groups. (**D**) The proportion of worse clinical outcomes after surgery is increased in the higher-CRG-score group. (**E**) The proportion of clinical outcomes in PC patients with high and low CRG scores after surgery. (**F**,**G**) Chemotherapeutic sensitivity for PC patients in low-CRG-score group. (**H**,**I**) Chemotherapeutic sensitivity for PC patients in high-CRG-score group. PR, partial response; PD, progressive disease; SD, stable disease; CR, complete response. The chi-square test was used to analyze the statistical differences between the two groups.

Previous studies have indicated that TMB is a valuable predictor of survival outcomes and immunotherapy response in tumor patients [23]. We explored the distribution alternations of somatic mutations between two CRG-score groups in TCGA cohort (Figure 6A,B). Patients with high CRG scores had substantially higher frequencies of TP53, KRAS, CDKN2, SMAD4, and TTN mutations than patients with low CRG scores, implying that these gene mutations were in charge of the poor prognosis of PC patients with high CRG scores. However, we observed opposite results regarding the mutation levels of RNF43, MUC16, and RYR1 (Figure 6A,B). In addition, our analysis of the mutation data demonstrated a higher TMB score in the high-CRG-score group compared with the low-CRG-score group (Figure 6C).

### 2.7. Clinical Outcomes and Drug Susceptibility Analysis

We investigated the CRG score’s ability to predict the impact of initial surgical treatment in PC patients. As displayed in Figure 6D,E, among the patients receiving the initial therapy of surgery, those with lower CRG scores showed significant treatment advantages.

Subsequently, to explore the efficacy of the CRG score as a biomarker for predicting chemotherapeutic susceptibility in PC patients, we assessed the semi-inhibitory concentration of 138 chemotherapeutic drugs commonly used to treat tumors. We identified 27 drugs more sensitive to patients with low CRG scores (Table S6), including EHT.1864 and PD.173074 (*p* < 0.01; Figure 6F,G). Nevertheless, 15 drugs responded better to patients with high CRG scores (Table S7), including paclitaxel and lapatinib (*p* < 0.01; Figure 6H,I). In brief, these findings suggest that the CRG score is associated with drug sensitivity.

### 2.8. Protein Expression Level of CAF-Related Risk Genes and Survival Analysis

**Figure 7:**
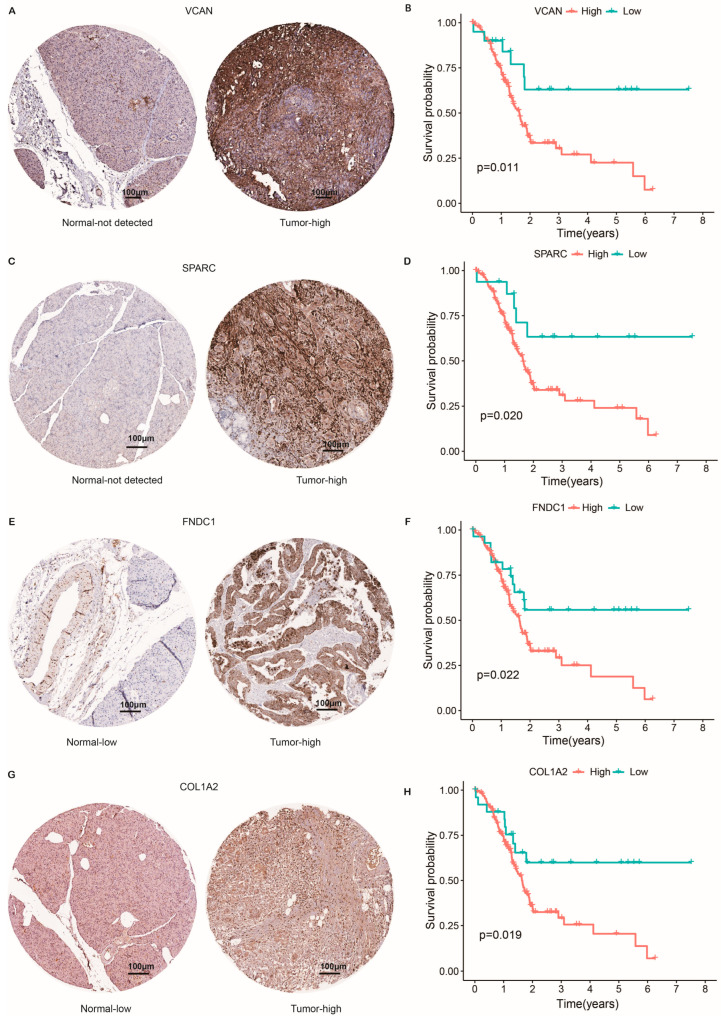
Immunohistochemical results of risk CRG expressions with their influences on OS. (**A**,**B**) The protein expression and survival of VCAN. (**C**,**D**) The protein expression and survival of SPARC. (**E**,**F**) The protein expression and survival of FNDC1. (**G**,**H**) The protein expression and survival of COL1A2.

To validate the tissue expression of risk CRGs in pancreatic normal and tumor tissues, we obtained immunohistochemical results from the Human Protein Atlas (HPA). Except for ZNF469, which is not available in the HPA database, consistent with the mRNA level in Figure 2B, protein expressions of VCAN (Figure 7A,B, *p* = 0.011), SPARC (Figure 7C,D, *p* = 0.02), FNDC1 (Figure 7E,F, *p* = 0.022), and COL1A2 (Figure 7G,H, *p* = 0.019) were higher in pancreatic tumor tissue, which is consistent with their correlation with the poor survival of PC patients.

## 3. Discussion

Immune and stromal cells, the essential TME components, are associated with the clinical features and prognosis of PC [24,25,26]. Extensive stromal involvement is a crucial hallmark of PC, which makes it challenging to obtain accurate tumor-specific molecular information [24]. Early studies identified that CAFs, a substantial portion of the tumor microenvironment, drove tumorigenesis and treatment resistance [6,27,28]. Previous studies revealed how CAF patterns affect the characteristics of TME and the efficacy of immunotherapy in triple-negative breast cancer (TNBC) [29]. With the development of tumor immunology and molecular biology research, immunotherapies, such as immune checkpoint inhibitors, have become new treatments for various tumors [30,31]. Recently, anti-PD-1/PD-L1 therapy has led to outstanding achievements in many malignancies [32,33]. However, due to the dense extracellular matrix acting as a physical barrier, PC patients remain poorly responsive to PD-1 antibodies [32,34]. Moreover, single-cell analysis also revealed that TGF-β-myCAF subtypes are related to the resistance to immunotherapy in breast cancer [35]. Whether analyzing CAF molecular subtypes improves the clinical response of PC remains to be determined [25]. Despite several studies having identified various biomarkers and clinical factors to predict PC prognosis [24,36], the relationship among CAF-based molecular signature, clinical outcome, and tumor microenvironment infiltration remains largely elusive in PC. Here, our study found an abundance of myCAF, apcCAF, and meCAF in the tumor tissues of PC. We also identified the alterations in genomic backgrounds and expression levels of CAF-related genes based on TCGA, GTEx, and ICGC cohorts. Most of the expressions of CRGs were increased in PC tumor tissues and correlated with prognosis. The aggregation of gene mutations leads to carcinogenesis, and gene mutations in PC may significantly impact immunotherapy response [37]. Among 25 CRGs, VCAN, FN1, and COL11A had the highest mutational intensity. However, there are currently no reports that these mutations are associated with carcinogenesis or fibrosis.

Additionally, we divided PC patients into two CAF clusters and observed discrepant prognoses, clinical characteristics, and immune infiltrations between them. The interaction of CAFs and immunity is a critical feature of tumorigenesis, which can serve as a therapeutic target for PC. Diverse CAF subsets play distinct roles in tumor immunosuppression of breast cancer. Their effects are achieved by Tregs regulating the proliferation of effector T cells [8]. Our findings showed that CAF cluster A, with a high enrichment of myCAF and apcCAF, had significantly higher stromal and immune scores than CAF cluster B. Cluster A also had higher infiltration levels of immunosuppressive cells, such as Tregs, MDSC cells, and DC cells, and other immunosuppressive factors, such as TGF-β-associated-ECM. Previous studies have shown that myCAF is the main component of the ECM [10]. Furthermore, apcCAF potentially modulates the immune response in pancreatic tumors [11]. Our results imply that CAF cluster A group may be closely associated with stromal activation and immunosuppression features, and myCAF and apcCAF abundance may be the main factors underlying such an immunosuppressive microenvironment. More interestingly, the higher enrichment score of anti-PD-1-resistant signatures and the lower enrichment score of nivolumab-responsive signatures were also observed in the CAF cluster A group, indicating that patients in the CAF cluster A group may be less sensitive to immunotherapy.

Furthermore, the CRG score was constructed to quantify CAF subtypes. The CAF subtype A with worse survival had higher CRG scores. Patients with higher CRG scores also had worse OS, implying that high CRG scores could predict an unfavorable prognosis. By integrating the CRG scores and clinical characteristics, we demonstrated that the CRG score was a unique, independent risk factor of OS. Moreover, we found a higher enrichment of meCAF in PC patients with low CRG scores. Our previous research showed that PDAC patients with abundant meCAF had a dramatically better response to immunotherapy [12]. Consistent with the previous conclusion, the CAF cluster B with better survival had a lower CRG score, and it also had a lower enrichment score of anti-PD-1-resistant signatures and a higher enrichment score of nivolumab-responsive signatures. These findings indicate that CRGs may participate in tumor immunosuppression; therefore, patients with low CRG scores can benefit from immunotherapy.

Due to PC patients’ distinctive molecular and clinical features, it is necessary to classify them precisely. We further identified potentially sensitive drugs in patients in different CRG-score groups. We expected that targeting CAFs combined with these drugs could reduce drug resistance and improve clinical outcomes.

## 4. Materials and Methods

### 4.1. Human Tissue Specimens

The Human Ethics Committee of Shanghai Renji Hospital, Shanghai Jiao Tong University School of Medicine (Shanghai, China), reviewed and approved research on human pancreatic cancer under informed consent from all patients.

### 4.2. Cell Lines and Mouse Pancreatic Cancer Model

Mouse pancreatic cancer cell lines KPC1199 were obtained from Jing Xue lab (Shanghai, China) and cultured in DMEM with 10% FBS. A total of 1 × 10^6^ KPC1199 cells were resuspended in 100 μL of PBS and injected subcutaneously into 6-week-old female C57BL/6 mice from Shanghai Laboratory Animal Center. The tumor tissues were ultimately weighed in 15 days and collected for flow cytometry analysis.

### 4.3. Masson’s Trichrome Staining

Formalin-fixed tissues were immersed in paraffin, and 5 μM sections were stained with Masson trichrome reagent to show collagen. First, the samples were partially dewaxed and rehydrated, fixed in Bouin’s liquor, and then washed and rinsed in distilled water overnight. Next, the slides were stained in Meyer hematoxylin solution for 5 min, and then placed in 0.5% hydrochloric acid and 70% ethanol for 5 s. After the specimens were washed three times and dissolved in 1% phosphomolybdic acid aqueous solution, the slides were stained with aniline blue or bright green for 5 min. Subsequently, we dehydrated the samples in 95% ethanol 10 times and added xylene to make them transparent. Finally, all slides were scanned and digitized using the digital pathological slice scanner system (Leica Biosystems Wetzlar, Germany). The collagen fibers were dyed blue, the nuclei were black, and the background was red.

### 4.4. Immunohistochemistry and Tissue Microarrays

Tissue microarrays included 91 PC samples. The histopathology of all cancer specimens was reassessed, and representative regions were labeled. Table S1 lists the prognosis information of individual patients in tissue microarrays. The immunohistochemistry staining was carried out to identify α-SMA and EpCAM marker expressions in tissue microarrays. As previously described, we conducted a semi-quantitative scoring system according to the distinct percentages of positively stained cells and staining intensity [38]. The frequency of positively stained cells was defined as 1+ (less than 25%), 2+ (25% to 50%), 3+ (50% to 75%), or 4+ (greater than 75%). Additionally, the intensity was scored as 0 (negative), 1+ (weak), 2+ (moderate), or 3+ (strong).

Finally, we multiplied the score of the positive area by the score of staining intensity to obtain the final immunohistochemistry score (range 0–12). We classified the 30 specimens in the tissue microarrays into five grades according to the above scores: 1+ (score 0), 2+ (score 1–2), 3+ (score 3–4), 4+ (score 6–8), and 5+ (score 9–12). The overall staining score of 5–12 was deemed high expression, while 0–4 was defined as low expression. The immunohistochemistry experiment used the following antibodies: anti-α-SMA rabbit polyclonal antibody (1:100, Cell Signaling, 3 Trask Lane, USA, #19245) and anti-EpCAM rabbit polyclonal antibody (1:100, Abcam, London, UK, ab223582).

### 4.5. Flow Cytometry Analysis

Briefly, the fresh tissue samples from two human PC tissues and seven mouse-derived allografts of KPC1199 cells were mechanically chopped and digested by collagenase IV at 37 °C for 30 min. The digested suspension was combined with DNase at room temperature for 5 min, washed twice with phosphate-buffered saline buffer containing 2% serum, and then filtered through the 100 μm filter. We used markers of CD29, PDPN, EpCAM, and CD45 to separate tumor epithelial cells (CD45^−^CD29^−^ or PDPN^−^EpCAM^+^), CAFs (EpCAM^−^CD45^−^CD29^−^ or PDPN^+^), and tissue leukocytes (CD29^−^ or PDPN^−^EpCAM^−^CD45^+^) in human and mouse tumor specimens, respectively. The digested single cells were washed twice and centrifuged for 5 min at 500× *g*, and then 1 μg/mL of antibody was added. Then, the samples were kept at 4 °C for 30 min in a dark place. Flow cytometry was employed using a BD Flow Cytometry Analysis Celesta cell sorter (Becton Dickinson, New York, NY, USA). The side-scatter width versus side-scatter region and the forward-scatter width versus forward-scatter height were applied to remove dead cells and cell clumps. Antibodies including anti-EpCAM-PerCR/Cy5.5 (BioLegend, San Diego, CA, USA, #324214), anti-CD45-APC-Cy7 (BioLegend, #368515), and anti-CD29-Alexa Fluor^®^ 488 (BioLegend, # 303015) were verified according to the manufacturer’s website.

### 4.6. PC Transcriptome Data Sources

The normalized transcriptome data, CNV files, somatic mutation data, and relevant clinicopathological and survival information of PC were acquired from the public database TCGA (https://xenabrowser.net/datapages/?cohort=TCGA%20Pancreatic%20Cancer%20(PAAD)&removeHub=https%3A%2F%2Fxena.treehouse.gi.ucsc.edu%3A443 (accessed on 1 June 2021)) and ICGC (https://dcc.ICGC.org/ (accessed on 1 June 2021)). In addition, the RNA expression data of normal pancreatic tissues were retrieved from the public database GTEx (https://xenabrowser.net/datapages/?cohort=GTEX&removeHub=https%3A%2F%2Fxena.treehouse.gi.ucsc.edu%3A443 (accessed on 1 June 2021). A total of 261 PC samples were acquired from TCGA (*n* = 160) and ICGC (*n* = 101) cohorts, and 167 normal pancreas samples were obtained from the GTEx cohorts. We excluded specimens from patients deficient in important clinicopathological or survival information.

### 4.7. Consensus Clustering Analysis of CRGs

Initially, 25 CRGs were identified from previous studies [24]. Then, we used the “ConsensusClusterPlus” package [39,40] to perform a consensus clustering analysis by the k-means algorithm to identify different CAF-associated subtypes. Furthermore, a protein–protein interaction (PPI) analysis through the string website (https://cn.string-db.org/ (accessed on 1 January 2022) was constructed to determine the interplay of CRGs.

### 4.8. Correlations of Molecular Patterns with the Clinical Features and Prognosis of PC

We explored the correlation of molecular subtypes, clinical variables, and survival outcomes to assess the clinical value of the two CAF subtypes. The clinical features included age (≥65 and <65 years), gender (male and female), tumor location (left and right side), TNM stage (stage I–IV), KRAS mutation status (abnormal and normal), and TP53 mutation status (abnormal and normal). In addition, the differences in OS between the two subtypes were estimated by Kaplan–Meier analysis using the “survival” and “survminer” packages [41].

### 4.9. Association of Molecular Subtypes with Tumor Immune Microenvironment of PC

We assessed PC patients’ immune and stromal scores using the ESTIMATE algorithm [42]. Then, the infiltrating fractions of 33 immune cell subtypes and four CAF subsets of each patient were computed with a single-sample gene set enrichment (ssGSEA) analysis algorithm [43].

### 4.10. Relationship of CAF Subtypes with Immunotherapy Responses in PC

As CAFs play a vital role in regulating tumor immune evasion [44], to further determine the association of CAF subtypes with immunotherapy responses of PC, we performed ssGSEA analysis to dissect the gene expression profiles of immunotherapy responses in the PC patients according to the nivolumab-responsive and anti-PD-1-resistant signatures [45].

### 4.11. Development of the CAF-Related Gene Risk Signature

We performed univariate and multivariate Cox regression analysis in TCGA-PAAD cohort to construct a novel CRG-based signature for predicting prognosis. Initially, the univariate Cox regression analysis was employed to assess the prognostic values of 25 CRGs in PC patients. Then, *p* < 0.05 was selected as a screening threshold, and 22 prognostic CRGs related to the survival of patients with pancreatic cancer were screened out. We further performed a multivariate Cox regression analysis and the penalized Cox regression model with the least absolute shrinkage and selection operator (LASSO) according to 22 prognostic CRGs. We obtained five hub prognostic CRGs (VCAN, COL1A2, ZNF469, SPARC, and FNDC1) and their corresponding coefficients. Finally, a scoring algorithm named CRG score was established to quantify the CAF state at the transcriptomic level. The CRG score was calculated as follows: CRG score = Σ (expression × correlative coefficient).

Additionally, we executed tROC curve analysis and independent prognostic analysis to validate the predictive capability of this novel CRGs-based signature and other clinical variables at 1-, 2-, and 3-year OS using the R package “survivalROC”.

### 4.12. Expression Level Validation of CAF-Related Risk Gene Expression

Immunohistochemical results of CRGs involved in the risk signature were obtained from the HPA database (https://www.proteinatlas.org/ (accessed on 1 January2022) to validate CRG expression in normal and tumor tissue.

### 4.13. Identification of Immune Microenvironment Affected by CRGs

We investigated the association between CRG score and immune microenvironment with several common methods, including XCELL, TIMER, QUANTISEQ, MCPOUNTER, EPIC, CIBERSORT-ABS, and CIBERSORT.

### 4.14. Correlation of CRG Score Signature with Signal Pathways, Tumor Mutation, and Chemosensitivity

To identify the differences in somatic mutations of PC patients between high- and low-CRG-score groups, the mutation annotation format was created with the “maftools” R package [46]. We further examined the dependence of the CRG score, clinical outcome, and TMB. Moreover, to explore diversities in chemotherapy drug efficacy between the two subgroups, we estimated the half maximal inhibitory concentration values (IC_50_) of chemotherapy drugs for each patient using the “pRRophetic” package [47], which is based on drug sensitivity data from the Genomics of Drug Sensitivity in Cancer dataset (https://www.cancerrxgene.org/ (accessed on 1 January2022)). A ridge regression model fitted the standardized expression data using predictor genes and the drug sensitivity (IC_50_) values as the outcome variables [48].

### 4.15. Additional Bioinformatics and Statistical Analyses

We applied the Wilcoxon test to analyze the inter-group differences, conducted Spearman analysis for correlation tests, and performed the log-rank and Kaplan–Meier tests to draw survival curves. The R 3.6.3 software (Bell Laboratories, New York, NY, USA) and its corresponding packages were used to process, analyze, and present the data. By comparing different groups, *p* < 0.05 was considered to indicate statistical significance.

## 5. Conclusions

In this study, we systematically analyzed the genomic backgrounds and expression levels of CRGs and inferred their latent role in PC patients’ prognosis and tumor microenvironment. We also constructed a novel CAF-associated gene signature as a robust biomarker to predict the prognosis, chemotherapeutic drug sensitivity, and immunotherapy impacts in PC. These results reveal the vital clinical significance of CRGs and put forward new ideas about the molecular classification of PC, which may be applied to precision medicine.

## Data Availability

The original contributions presented in the study are included in the article. Further inquiries can be directed to the corresponding author.

## Supplementary Materials

The following supporting information can be downloaded at https://www.mdpi.com/article/10.3390/ijms24010156/s1.
