# Supplementary material for: A Novel Molecular Signature of Cancer-Associated Fibroblasts Predicts Prognosis and Immunotherapy Response in Pancreatic Cancer
Source: Int J Mol Sci. 2022 Dec 21;24(1):156. doi: 10.3390/ijms24010156 (PMC9820557; doi:10.3390/ijms24010156)
Supplement: Supplementary file 1 [file ijms-24-00156-s001.zip › Supplementary figure and table legends.pdf]

Supplementary figure and table legends

Supplementary Figure S1. Identification of the most suitable k value to distinguish CAF cluster by consensus cluster analysis.

Supplementary Figure S2. Validation of the two different CAF subtypes in different series. (A-B) Consensus matrix heatmap defining two CAF clusters (k = 2) in the ICGC cohort. (C) Kaplan-Meier survival curve showed significant differences in OS between the two CAF clusters in the ICGC cohort (log-rank test, P=0.007). (D) Gene set variation analysis analyzed the biological pathways of two CAF subtypes in PC samples from TCGA and ICGC cohorts. CAF cluster, tumor location, stage, gender, age, KRAS mutation, TP53 mutation, and survival status were used as sample annotations.

Table S1. Prognostic information of 91 PC tissue microarrays (TMA).

Table S2. Detailed information on the 160 PC patients from the TCGA cohort.

Table S3. The comprehensive landscape of CAF interactions, regulator connections, and their prognostic values in PC patients.

Table S4. GSVA enrichment analysis of the CAF subtypes in TCGA and ICGC cohort.

Table S5. The correlations between the two CAF subtypes and 33 immune cell subsets of PC samples.

Table S6. 27 highly susceptible drugs in patients with low CRGs score.

Table S7. 15 highly susceptible drugs in patients with high CRGs score.
