# Supplementary figures and images for: A Novel Molecular Signature of Cancer-Associated Fibroblasts Predicts Prognosis and Immunotherapy Response in Pancreatic Cancer
Source: Int J Mol Sci. 2022 Dec 21;24(1):156. doi: 10.3390/ijms24010156 (PMC9820557; doi:10.3390/ijms24010156)

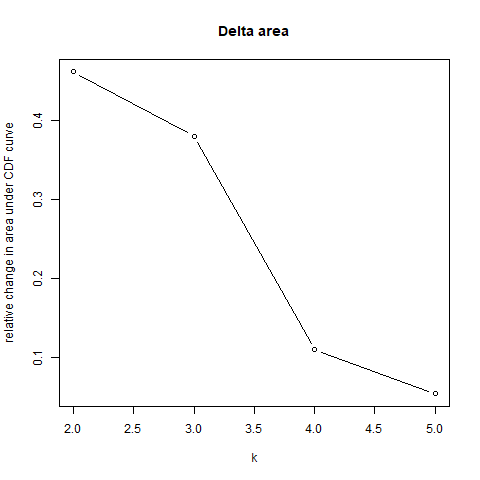

Supplement: Supplementary file 1 [file ijms-24-00156-s001.zip › supplemental figure S1.png]

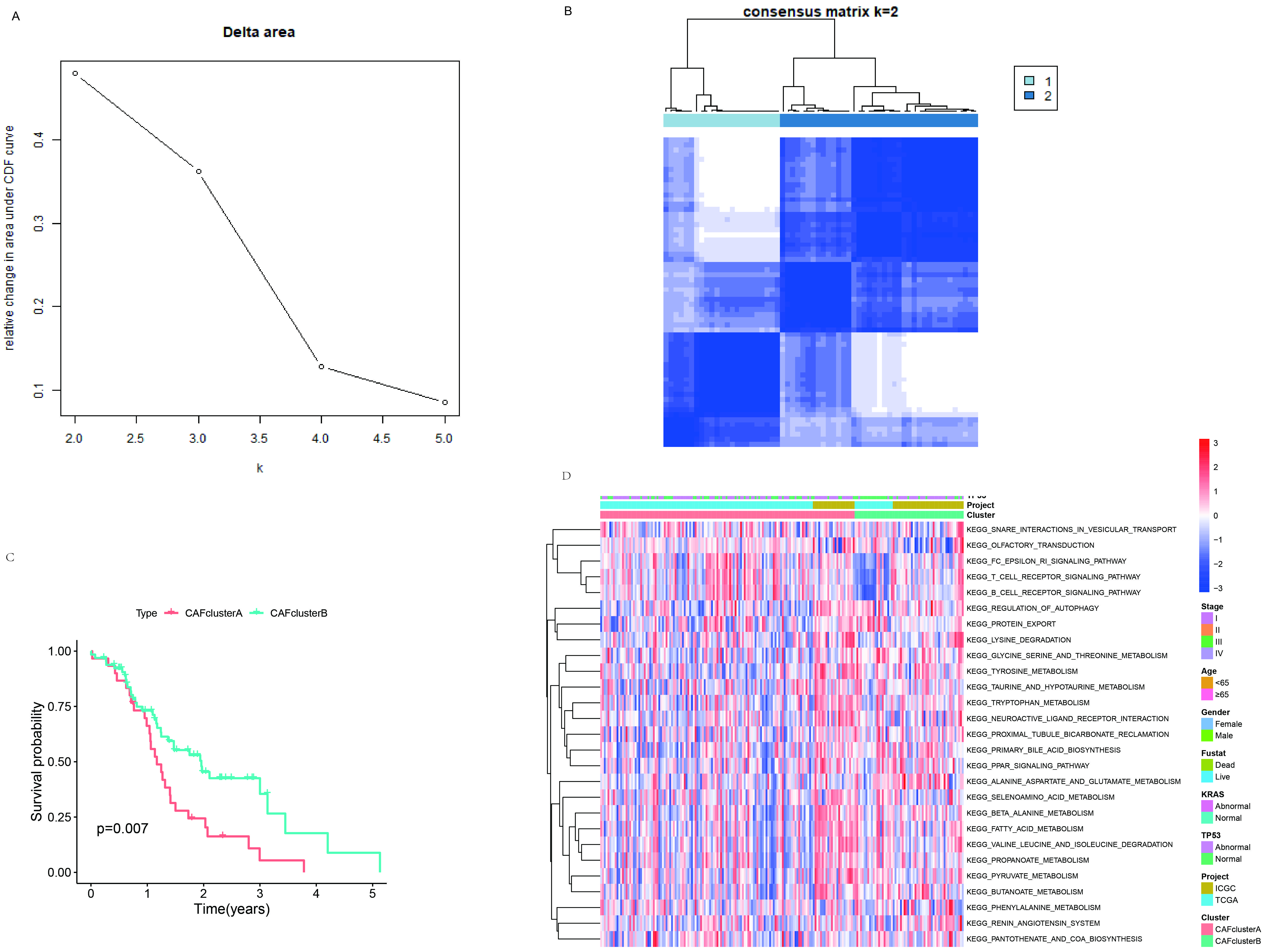

Supplement: Supplementary file 1 [file ijms-24-00156-s001.zip › Supplemental figure S2.jpg]
